# The closure of the Vardar Ocean (the western domain of the northern Neotethys) from the early Middle Jurassic to the Paleocene time, based on the surface geology of eastern Pelagonia and the Vardar zone, biostratigraphy, and seismic-tomographic images of the mantle below the Central Hellenides

**DOI:** 10.14324/111.444/ucloe.000024

**Published:** 2021-09-22

**Authors:** Rudolph Scherreiks, Marcelle BouDagher-Fadel

**Affiliations:** 1 Geologische Staatssammlung of the Bayerische Staatssammlung für Palaeontologie und Geologie, Luisenstr. 37, 80333 Munich, Germany; 2 University College London, Office of the Vice-Provost (Research), 2 Taviton Street, WC1H OBT, London, UK

**Keywords:** Adria, Pelagonia, Vardar, subduction, obduction, tectono-stratigraphy, biostratigraphy, tomographic images, ophiolite, carbonate platforms, ocean lithosphere

## Abstract

Seismic tomographic images of the mantle below the Hellenides indicate that the Vardar Ocean probably had a composite width of over 3000 km. From surface geology we know that this ocean was initially located between two passive margins: Pelagonian Adria in the west and Serbo-Macedonian-Eurasia in the east. Pelagonia was covered by a carbonate platform that accumulated, during Late Triassic to Early Cretaceous time, where highly diversified carbonate sedimentary environments evolved and reacted to the adjacent, converging Vardar Ocean plate. We conceive that on the east side of the Vardar Ocean, a Cretaceous carbonate platform evolved from the Aptian to the Maastrichtian time in the forearc basin of the Vardar supra-subduction volcanic arc complex. The closure of the Vardar Ocean occurred in one episode of ophiolite obduction and in two episodes of intra-oceanic subduction. **1.** During the Middle Jurassic time a 1200-km slab of west Vardar lithosphere subducted beneath the supra-subduction, ‘Eohellenic’, arc, while a 200-km-wide slab obducted onto Pelagonia between the Callovian and Valanginian times. **2.** During the Late Jurassic through to the Cretaceous time a 1700-km-wide slab subducted beneath the evolving east Vardar-zone arc-complex. Pelagonia, the trailing edge of the subducting east-Vardar Ocean slab, crashed and underthrust the Vardar arc complex during the Paleocene time and ultimately crashed with Serbo-Macedonia. Since the late Early Jurassic time, the Hellenides have moved about 3000 km toward the northeast while the Atlantic Ocean spread.

## Introduction

**Figure 1:**
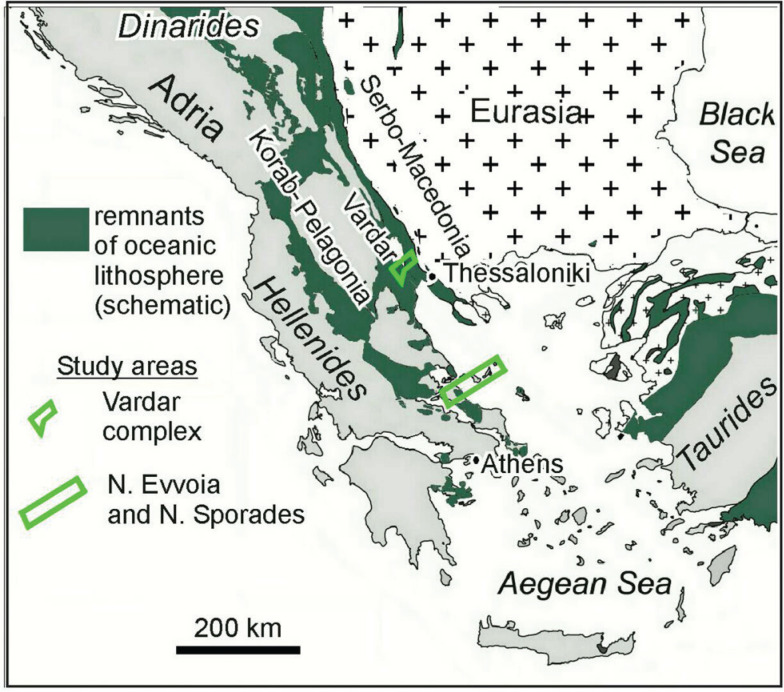
Neotethys oceanic lithosphere in the Dinarides through the Hellenides and Taurides, represent remnants of the northern branch of the Neotethys (altered after Ustaszewski et al. [12]). Our study areas are in Evvoia and the Northern Sporades, and in the ‘Vardar zone’ of Greek Macedonia. Fieldwork was carried out in the Vardar zone and Northern Evvoia in September and October 2020 and Evvoia and the Northern Sporades in previous years.

Relicts of oceanic lithosphere can be traced from the Dinarides through the Hellenides and Taurides. They bear witness to the once extensive northern Neotethys Ocean (Fig. 1) [1–3]. In this paper, we shed new light on the paleogeography and subduction of the Vardar branch of the Neotethys Ocean from the Early Jurassic through the Early Paleocene time, which we have gained from our research on the tectono-stratigraphy of the Vardar zone of Greek Macedonia and from the eastern Pelagonian zone of Northern Evvoia and the Northern Sporades (Fig. 1). This surface geology is aligned with seismic tomographic images that depict two perturbations in the mantle below the central Hellenides, that we interpret as two slabs of Vardar Ocean lithosphere, which sank into the mantle during two episodes of subduction. We also show that two carbonate platforms evolved, one on each side of the Vardar Ocean and they reacted to and were tectonically involved with the obduction, subduction and ultimate closure of the Vardar Ocean.

A time-lapse reconstruction is presented of the convergence and subduction of the Vardar Ocean from the Early Jurassic through the Early Paleocene time. We give answers to questions concerning the original width of the Vardar Ocean and how closure took place and ended with Pelagonia’s collision with the Vardar Island-arc complex and the detachment and subsidence of the Vardar Ocean slabs into the mantle.

## Geological background

### The Neotethys, Vardar zone and some nomenclature

**Figure 2:**
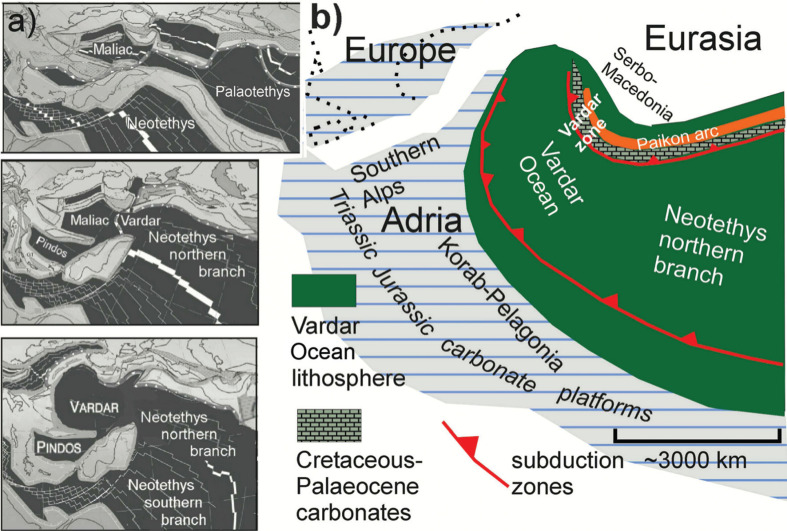
Paleogeography and evolution of the Vardar Ocean (a) altered after Stampfli and Borel [1]; (b) altered after Schmid et al. [2]; Gallhofer et al. [85] and Van Hinsbergen et al. [86], in Schmid et al. [3]. a) The Vardar domain of the Northern Tethys Ocean evolved out of the Maliac and Paleotethys in the Permo-Triassic time. b) The Vardar Ocean was situated between continental Adria (including Korab-Pelagonia) and Serbo-Macedonian Europe. The paleogeography implies that early Middle Jurassic intra-oceanic subduction led to the obduction of the Eohellenic ophiolite onto eastern Pelagonia and, subsequently, that Vardar Ocean lithosphere subducted beneath the Paikon island arc and led to the collision of eastern Pelagonia with the island arc. See text.

In paleogeographical reconstructions of the evolution of the Palaeotethys and Neotethys, Stampfli and Borel [1] show that the northern Neotethys Ocean opened as the Palaeotethys closed (Fig. 2a): the Maliac Ocean is a remnant of the Paleotethys, which, through intra-oceanic subduction, becomes overthrust by the Vardar Ocean at the western end of the northern Neotethys. Alternatively, the Vardar Ocean can simply be envisioned to have opened as a western continuation of the Neotethys (Sengör and Natal’in [4] in Hafkenscheid [5]).

In an enlightening palaeogeographical reconstruction of the mid-late Jurassic Vardar Ocean, shown in Schmid et al. [3] the Vardar Ocean has two eastward dipping, intra-oceanic subduction zones and an arc complex (Fig. 2b). This model implies that the Vardar Ocean existed from the Early Mesozoic to the Late Cretaceous time (in agreement with Sharp and Robertson [6]). Our research corroborates these plate-tectonic paleogeographical interpretations which we have proceeded to investigate both spatially and temporally. Following Schmid et al. [2] the present contribution supports the one-ocean concept, that the Vardar ophiolites were obducted westward over the Korab–Pelagonian zone of east Adria (Fig. 2b). For other models in which western Pelagonia had plate-tectonic involvement with an inferred Pindos Ocean see Sharp and Robertson [6]. Our investigations, however, have been limited to eastern Pelagonia and the Vardar zone (Fig. 1).

#### Nomenclature

For nomenclatural orientation, ‘Vardar Ocean’ is the name of the western ocean domain of the northern Neotethys (Fig. 2b). We agree with Schmid et al. [3] that ‘Vardar zone’ (Fig. 2b) is not synonymous with ‘Vardar Ocean’. In our opinion, the Vardar zone is not the ‘root’ of Vardar-derived thrust sheets, as has been often suggested [7–9]. Quite the contrary, as will be shown, the ‘Vardar zone’ is where the last slab of the Vardar Ocean was subducted [10,11] and probably corresponds to the ‘Sava suture zone’ [3,12].

The names of geo-tectonic sub-divisions of the Vardar zone used herein are after Kockel [13].

The ‘Vardar zone’ corresponds to the northwest–southeast striking belt (Fig. 1) where remnants of island arc volcanic formations are found [6,14–18,23] and where easternmost Pelagonia is covered by Upper Cretaceous carbonates [3].

We consider it important to use the term ‘ophiolite’, in the strict sense of the ‘Steinmann Trinity’ [19], because there are oceanic formations in the study areas that are composed of basalt +- radiolarite but are devoid of serpentinite and were derived from tectonic environments unrelated to obduction, which will be shown.

Furthermore, the term ‘mélange’, used herein, follows Hsü [20] referring to tectonically produced polymictic fault-zone rocks as opposed to polymictic sedimentary deposits (see also Scherreiks [21]). The mélanges are associated with mylonitic S–C shear fabrics of subduction zones [22] like those found in the Vardar zone [23].

### The carbonate platforms of Adria and the Vardar zone

Following the aforementioned and our own research, Adria was the fundamental pedestal of a vast subsiding carbonate platform, of the marginal, foreland category [24–26] that extended from the Alps [27,28] through Korab-Pelagonia and into the west Taurides [21,29,30] (Figs 1 and 2b) and across the western Adria [31]. The platform evolved adjacent to the west side of the Vardar Ocean during the Late Triassic through the Early Jurassic from a cyclically alternating supratidal to a peritidal domain [21,32] and then responded with subsidence and episodes of upheaval as continental Adria and the Vardar Ocean converged [33–35]. (Table 1a documents biostratigraphic data concerning the Pelagonian carbonate platform of Evvoia and the Northern Sporades, which will be referred to in the text.)

**Table 1a.** Biostratigraphic data, Evvoia and the Northern Sporades

Biostratigraphy of Evvoia and Northern Sporades [10,21,33,34,81]
**Pelagonian carbonate platform** **1. Rhaetian–Hettangian: peritidal/subtidal** ? *Aulotortus* sp., ‘*Aulotortus friedli*’, *Auloconus permodiscoides, Grillina* sp.‘*Vidalina*’ *martana* **2**. **Sinemurian–Early Pliensbachian: shallow warm reef environment** *Siphovalvulina colomi, Siphovalvulina gibraltarensis, Duotaxis metula, Lituosepta recoarensis, Riyadhella praeregularis. Lituosepta compressa, Riyadhella praeregularis, Palaeodasycladus mediterraneus, Pseudocyclammina liasica, Lituosepta recoarensis* **3. Aalenian–Bathonian: shallow water environment** *Mesoendothyra croatica* Gusîc´ **4. Middle to Upper Jurassic: shallow water environment** [81] *Neokilianina rahonensis*
**5. Bathonian–Callovian foraminifera suite: shallow warm reef environment This limestone occurs below the below the bauxite** *Pseudomarssonella bipartita*, *Redmondoides medius*, *Andersenolina elongata, Riyadhella* sp., *Ammobaculites* sp., *Trocholina* sp., *Palaeodasycladus* cf. *mediterraneus*, *Pseudopfenderina* sp., *Everticyclammina* sp., *Siphovalvulina* sp., *Riyadhoides* sp. **6. Callovian–Oxfordian foraminifera suite on top of laterite: shallow reef environment** *Chablaisia* sp., *Septatrocholina banneri*, *Andersenolina elongata*, *Andersenolina* sp., *Palaeodasycladus* sp.
**7. Upper Jurassic shallow patch-reef environment** *Protopeneroplis striata*, *Parurgonina caeinensis*, *Thaumatoporella parvovesiculifera, Actinostromaria tokadiensis* **8. Late Berriasian–Early Valanginian: shallow reef environment** *Cladocoropsis mirabilis*, *Zergabriella embergeri*
**9. Late Cretaceous transgression in Evvoia, Maastrichtian: outer neritic environment** *Plummerita* aff. *hantkeninoides, Idalina* aff *antiqua*, Hippurites sp., *Planorbulina cretae*: on a rudist clast (Campanian).
**Cretaceous carbonate platform of the Northern Sporades** **10.1 Albian to Santonian: shallow reef environment** *Nezzazatinella picardi*, *Nezzazata convexa, Dicyclina schlumbergeri* **10.2 Late Santonian to Maastrichtian: reef/forereef environment** *Rotorbinella* sp., *Orbitoides* sp., *Lithocodium* sp., *Lithocodium aggregatum*, rudists **10.3 Early Paleocene: shallow reef environment** *Kathina* sp., *Daviesina* sp., *Lockhartia* sp.
**Radiolarians in Evvoia** **11. Ophiolite sheet:** Scherreiks et al. [34], determined in co-operation with P. O. Baumgartner, Gingins and Schauner [88]. **11.1 Carnian to Lower Norian:** *Annulotriassocampe* ? sp., *Castrum* ? sp., *Corum* ? sp., *Capnuchosphaera* cf. *crassa Capnuchosphaera* sp. **11.2 Elias complex, Middle to Late Jurassic:** Spongocapsula hooveri, Parvicingula dhimenaensis s.l. Transhuum brevicostatum, Protunuma sp., Sethocapsa sp.
**12. Ophiolite mélange** [36,38] **Middle Bathonian to Lower Callovian** *Parvicingula dhimenaensis* ssp., *Mirifusus fragilis* s.l., *Transhsuum maxwelli* gr., *Tricolocapsa plicarum* s.l.

In the Vardar zone at the east side of the Vardar Ocean (Fig. 2b) one finds the remnants of a carbonate platform that evolved during the Cretaceous, most probably on the forearc margin of the Vardar arc (Fig. 2b) whose evolution terminated during the Paleocene [14,17]. The inevitable crash between Pelagonia and the Vardar zone (Fig. 2b) was a collision between two Cretaceous platforms (see Discussion and conclusions). (Significant biostratigraphic data concerning carbonate platform of the Vardar zone are documented in Table 1b and 1c and will referred to.)

**Table 1b.** Biostratigraphic data, west and central Almopias

**West and Central Almopias** After Mercier and Vergely [55] Updated and additional age and palaeoenvironmental determinations [89,90]
**1. West Almopias** **1.1 Late Maastrichtian (Maastr, 2): inner neritic environment** Planktonic foraminifera *Abathomphalus mayaroensis, Globotruncana Stuarti, Contusotruncana contusa, Globotruncana arca* and *Globotruncana linneiana* and the larger benthic foraminifera *Orbitoides medius* **1.2 Santonian–early Campanian: shallow reef/intertidal environments.** The Hippuritidae, Vaccinites atheniensis
**2 Kato Grammatiko Pyrgi: Cenomanian (Cen. 1): forereef/inner neritic environment.** Planktonic foraminifera *Rotalipora appenninica* and larger benthic foraminifera *Nezzazata simplex*
**3. Kerassia Campanian-Maastrichtian (Camp. 3b–Maast 2): inner to outer neritic environment** *Globotruncana arca* [= *G. convexa*], *Globotruncanita* gr. *struarti-stuartiformis*
**4 Kerassia–Nisi–Kedronas** **4.1 Campanian (3, 77.0-72.1 Ma): Inner to outer neritic planktonic foraminifera in micritic wackestone:** *Radotruncana subspinosa*; *Heterohelix dentata*, *H.* spp.; *Globotruncana lapparenti*, *G. aegyptiaca*, *G. ventricosa*, *G. linneiana*, *G. rosetta*, *G. arca*; Co*ntusotruncana fornicata*; *Ventilabrella glabrata*; *Rugoglobigerina rugosa*, *R. hexacamerata*; *Globotruncanita atlantica*, *Gl. stuarti*, *Gl*. sp.; *Schackoina* sp.; *Globotruncanella* sp.; *Archaeoglobigerina blowi*. **4.2 Aptian (Apt. 1**–**4a): reefal to inner neritic environment depositional depths of between 10 and 50 m.** The presence of the larger benthic foraminifera *Palorbitolina discoidea* Gras (Barremian to Aptian), Palorbitolina lenticularis, indicate Aptian 1–4a age 125–115 Ma (see BouDagher-Fadel and Price [91]).
**5. Kerassia–Kedronas–Kato Grammatiko Campanian-Maastrichtian (Camp. 3**–**Maast): reefal (rudist debris) to reworked in outer neritic** Globotruncana *arca*, *Globotruncanita stuarti*, *Globotruncana linneiana* [= *G. tricarinata*] **5.1 Late Santonian (Sant.2): outer neritic** *Globotruncana lapparenti*, *Globotruncana arca* [= *G. convexa*], Marginotruncana coronata, *Sigalia deflaensis* **5.2 Early Santonian (Sant. 1): outer neritic** *Praeglobotruncana turbinata*, *Sigalitruncana sigali*, *Marginotruncana coronata*, *Globotruncana linneiana Globotruncana lapparenti*.
**6 Jurassic exposures in the Kerassia**–**Nisi area (Pelagonian origin) Oxfordian–Early Cretaceous: low energy environment** *Stylosmilia* cf. *miehelini*, *Thecosmilia* cf. *langi*, *Cladocoropsis mirabilis*, *Dermosmilia* sp. and *Schizosmilia* cf. *rollieri* indicate a? Late Oxfordian–? Early Kimmeridgian age (in Sharp and Robertson [6])
**7. Central Almopias** (**Maragarita and Klissochori** limestones on top of Jurassic mélange) with ‘conglomeratic’ lenses **7.1 Flamouria, (east of Edessa) Early Santonian: outer neritic** *Marginotruncana coronata, Globotruncana arca* [= *G. convexa*, *Marginotruncana marginata*. The shallow water Early Cretaceous. larger benthic foraminifera, *Orbitolina* sp. are reworked into the pelagic assemblages **7.2 Messimeri (beneath Central Almopias mélange south of Edessa)** *Cladocoropsis* sp. indicates Late Jurassic age and Pelagonian

**Table 1c.** Biostratigraphic data, east Almopias and Paikon

**East Almopias and Paikon** (after Mercier and Vergely [59]) updated age and environment [89]
**1. Nea Zoi** **1.1 Cenomanian (Cen. 3): outer neritic environment.** *Rotalipora cushmani* and *Praeglobotruncana stephani* **1.2 Late Santonian**–**early Campanian (Sant. 2–Camp. 2): inner to outer neritic** *Globotruncanita elevata*, *Globotruncana convexa*, Globotruncana arca, *Orbitoides media*
**?2. Krania-Mavrolakkos Unit.** Radiolarian determinations (P. De Wever and H. YiLing; in Sharp and Robertson [6,65]) ages ranging from Callovian to Early Cretaceous?
**3. Krania Unit: Mid-Oxfordian to Valanginian** Radiolarians [63]
**4. Vryssi Unit and Nea Zoi Unit:** basalts are overlain by radiolarite of **Late Triassic** [63]
**Paikon** **5. Theodoraki unit** **5.1 Late Maastrichtian (Maast. 2**–**3): outer neritic** *Globotruncana linneiana, Contusotruncana contusa*, *Globotruncana arca* **5.2 Maastrichtian (Maast. 2**–**3): outer neritic** *Globotruncana arca* [= *G. convexa*], *Globotruncana linneiana* [= *G. tricarinata] Globotruncana calciformis*, *Contusotruncana contusa* indicate late Maastrictian age. **5.3 Early Campanian (Camp 1**–**2): outer neritic** *Globotruncanita stuartiformis* indicates Campanian Santonian *Marginotruncana marginata* indicates an early Santonian age reworked into early Campanian assemblage **5.4 Earl Cenomanian (Cen. 1): reef/inner neritic** *Orbitolina* gr. *Concava, Nezzazata* sp., *Cuneolina* sp, *Cycloloculina* sp., *Pseudolituonella* sp. (see BouDagher-Fadel [81])
**6. Griva-Khromni mélange** (from numerous researchers in Katrivanos et al. [23]) **6.1 Aptian**–**Early Albian** *Mesorbitolina* sp., *Sabaudia minuta* **6.2 Late Jurassic to Early Cretaceous** *Actinoporella* sp., *Pseudocyclamina* sp., *Cuneolina* sp., *Cladocoropsis mirabilis*, nerineid gastropods

#### The Pelagonian carbonate platform and its involvement in the demise of the Vardar Ocean

The Vardar Ocean existed during the Middle to Late Triassic, substantiated by radiolarians and pillow basalt found in ophiolite occurrences in our study area in Evvoia [33,36–40] (Table 1a 11.1). Initially, the late Triassic and early Jurassic carbonate platform evolved from a cyclically alternating supratidal to peritidal domain [21,32] and then began sinking, presumably responding with subsidence as Adria converged with the Vardar Oceanic plate [33]. The postulated beginning of intra-oceanic obduction was around the Toarcian to Bajocian time (180–170 Ma), based on the ages of amphibolites found in the ‘metamorphic sole’ of subduction-zone mélanges [41–43]. The platform subsided during the Middle Jurassic, verified by ever deepening carbonate facies [21], and then became emergent during the Callovian time, verified by bauxite deposits (Fig. 4a) [35]. The age of this Callovian upheaval has been verified with Bathonian foraminifera in the limestones below, and Oxfordian foraminifera above the bauxite crusts (Table 1a 5 and 6) [55]. The ‘Callovian event’ has been attributed to plate tectonic stress that affected the entire Mediterranean region [44]. An Oxfordian transgression re-established shallow marine environments which generated a Tethys-wide reef facies that extended from the Alps to Asia and in the Hellenides is characterised by the demosponge, *Cladocoropsis mirabilis* Felix [21,29] (Table 1a 7 and 8). Rapid platform subsidence and drowning below the carbonate compensation depth (CCD) occurred during the Tithonian–Berriasian time, verified by radiolarian cherts [45]. The final ophiolite emplacement is estimated to have occurred in the Valanginian time, in Evvoia, after flysch-like sedimentation had been shut off by the obduction [21,33,34]. The obduction was followed by a period of ophiolite erosion (post-Eohellenic unconformity: Scherreiks [21]) and a subsequent gradual, widespread, transgression of marine conglomerate in Evvoia and across the Pelagonian zone during the Early Cretaceous time [21,46,47] (Table 1a 9).

### Paleogeography of the Vardar Ocean discerned from seismic tomographic images of the mantle below the Hellenides

Seismic tomographic images of the Alpine–Himalayan realm (BSE models, Bijwaard et al. [48]) depict mantle-perturbations of subducted slabs of the Neotethys oceanic lithosphere [5,49,50].

Van Hinsbergen and others [51] recognised two separate and distinct perturbations in tomographic images as probable Neotethys slabs.

**Figure 3:**
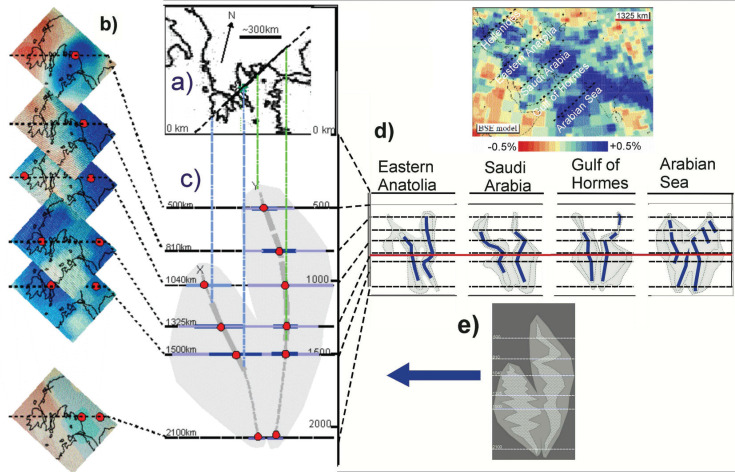
Seismic tomographic images below the Central Hellenides. a) Map sketch of the Hellenides shows the position of the NE–SW vertical section through the mantle below the Central Hellenides c). b) Seismic tomographic images (BSE models, ascertained from Hafkenscheid [5]) of horizontal sections through the mantle at six different depths. They depict contours of seismic velocity anomalies (see Hafkenscheid [5] for the theoretical background). c) The vertical section through the BSE models. The sketch schematically depicts perturbation ‘clouds’ containing the lithospheric ‘slabs’ (see e). Slab x has sunk about 900 km, slab y has sunk about 400 km. d) Vertical sections depicting the mantle eastwards of the Hellenides show that there are two sinking lithospheric slabs. Positions of sections are shown in BSE Model 1325 km. e) The perturbations appear to bulge with depth l, suggesting that subducted slabs undergo vertical compression and folding(?) in which case, only the minimum widths of the original slabs can be estimated.

For our investigations, we have enlarged the tomographic images of the areas below the Hellenides and have discerned that there are two slabs: the western slab has a width of about 1200 km, while the eastern slab has a width of about 1700 km, added together to make 2900 km (Fig. 3a–c). To check this, we looked further eastwards to the Arabian Sea (Fig. 3d) and have corroborated that two slabs of oceanic lithosphere have also been subducted there. Figure 3d, in detail, is highly interpretive; however, two distinct parallel perturbations are apparent. We have interpreted the perturbations beneath Hellenides as sunken Vardar Ocean lithosphere and are of the opinion that the images verify two episodes of subduction [10] (Fig. 3c) (see Discussion and conclusions).

## The study areas

### Evvoia and Northern Sporades

#### Ophiolites and platforms

**Figure 4:**
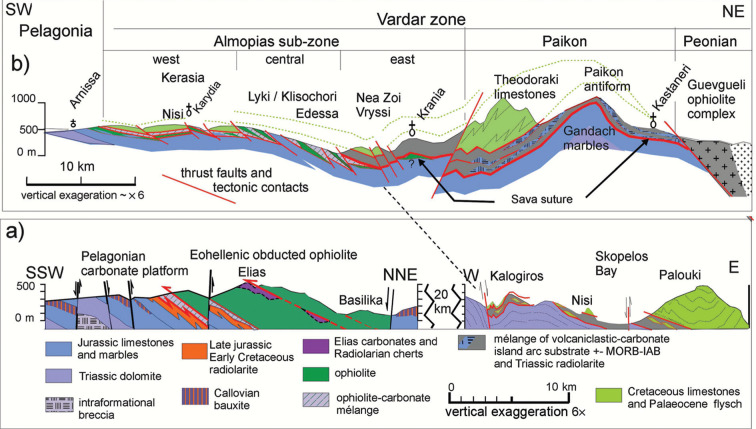
Overview tectonic sections of the study areas (nomenclature ‘Almopias, Paikon and Peonian’ units after Kockel [13]). a) Western part of section shows obducted ophiolite, composed of serpentinite, peridotite, basalt, gabbro and radiolarian chert, which was obducted together with tectonic mélange over the Pelagonian carbonate platform [21]. The Elias formation has been interpreted as a relict of a supra-subduction island arc complex [34]. Bauxite was deposited during the Callovian [35] (Table 1a 5 and 6). The eastern part of section a) shows overthrust, supposed Vardar, Cretaceous platform carbonates and mylonitised ocean floor mélange (devoid of serpentinite). This nappe overlies the post-Eohellenic erosional unconformity of Upper Triassic dolomite [10]. b), shows the Vardar zone between the Guevgueli ophiolite complex and Pelagonian ophiolite near Arnissa. Exposures of Pelagonia-derived ophiolite s. str. occur in the western and central parts of the Almopias zone near Karydia and Lyki/Klisochori; Serpentinite is not found in the units of the Paikon sub-zone (see also Fig. 6).

Examples of obducted ophiolite s. str. occur in the study areas of northern Evvoia (Fig. 4a) [21,34] and are found throughout the Korab–Pelagonian zone (Fig. 1). They lie, tectonically emplaced, together with mélange on top of Upper Jurassic and Lower Cretaceous carbonate platform rocks [52,53]. The ophiolites are erosional remnants that have been postulated to be parts of a single obducted ophiolite sheet that was emplaced during the Late Jurassic to Early Cretaceous, an age which classifies it as ‘Eohellenic’ after Jacobshagen et al. [52]. The onetime ophiolite sheet is considered to have had a width of at least 200 km – when judged from the width of the ophiolite outcrops on geological maps [3,39] (Fig. 1).

**Figure 5:**
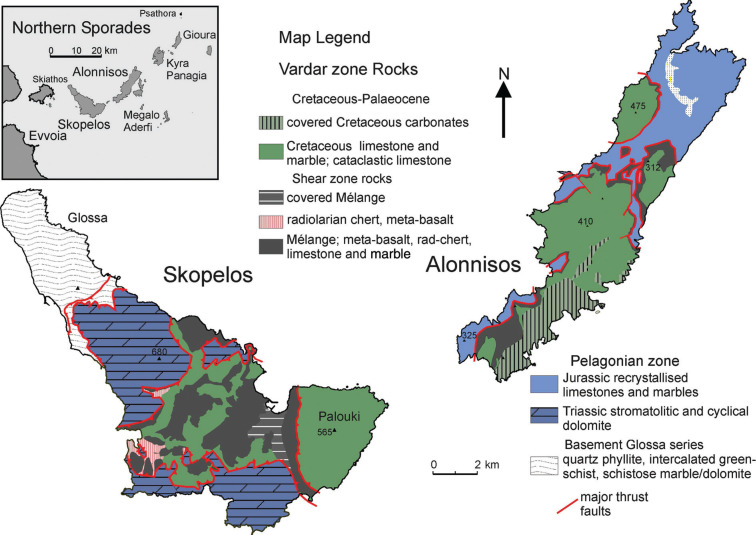
Overview geological map of Skopelos and Alonnisos in the Northern Sporades (based on Matarangas [87]; Kelepertsis [54] and Scherreiks and BouDagher-Fadel [10]). The Cretaceous limestone formation of Alonnisos and Skopelos lies tectonically emplaced, together with a sheared mélange of metamorphic ocean-floor basalt and radiolarian chert, on top of the post-Eohellenic erosional unconformity over Pelagonian Upper Jurassic limestone on Alonnisos and Upper Triassic dolomite on Skopelos. It has been postulated that the tectonic emplacement took place during Paleocene time as Pelagonia underthrust the Cretaceous forearc basin of the Vardar volcanic arc [10].

The Northern Sporades are devoid of serpentinite. The ophiolite sheet, known to have been obducted over Pelagonia, had been eroded from large areas of Pelagonia during the later Lower Cretaceous time (see above). On the Sporades, erosion was extreme; the ophiolite and large parts of the carbonate platform are missing (Fig. 5). The eroded surface of Jurassic and Triassic platform carbonates is covered by a sheet of mélange composed of meta-basalt and radiolarian chert which is chaotically mixed with carbonate breccia and mylonitic phyllonites [10] (Figs 4a and 5). Slices of Cretaceous and Paleocene platform carbonates of reefal origins are tectonically incorporated in the melange (Table 1a 10–10.3). The Cretaceous carbonate platform successions of Alonnisos and Skopelos overlie the mélange. In corroboration with Kelepertsis [54] we suggest that the Cretaceous and Paleocene carbonates of the northern Sporades are of Vardar zone origin, which will be expanded upon in the Discussion and conclusions. The Cretaceous carbonate platform and its mélange substrate, we suggest, correlate with a similar succession in the Almopias sub-zone (Fig. 4a and b).

### The Vardar zone

#### West Almopias and its tectonic contact with Pelagonia

**Figure 6:**
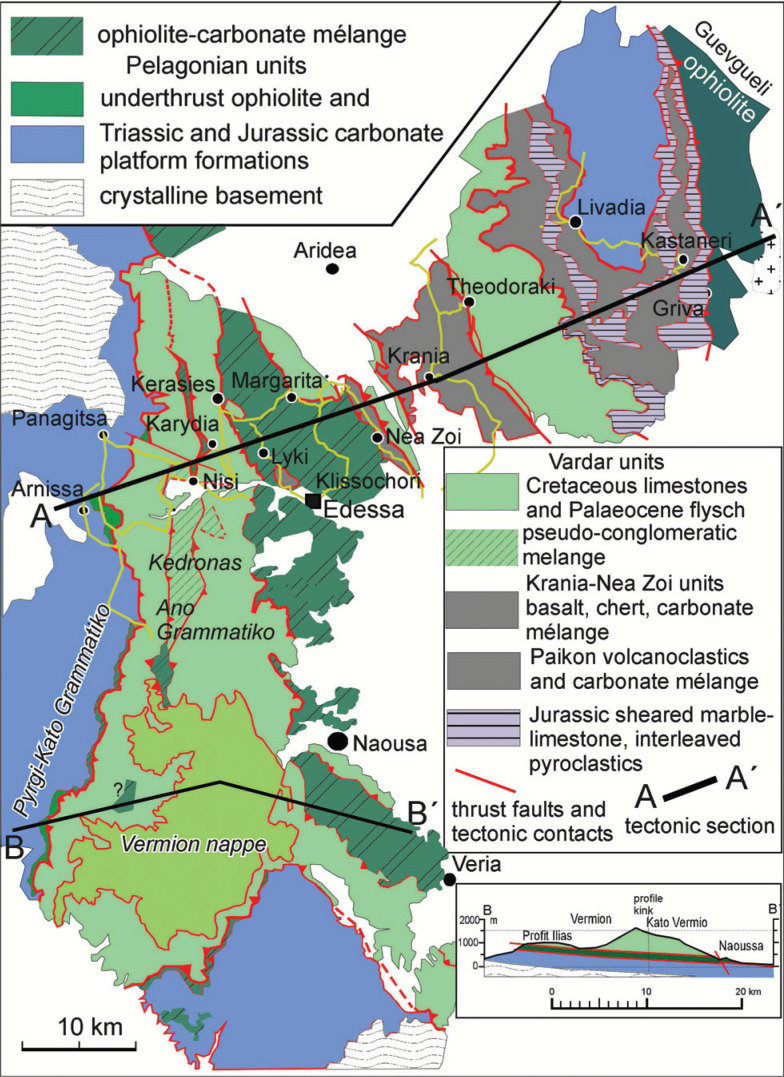
Geological overview map of the Vardar and adjacent Pelagonian zone (based on Mercier and Vergely [55] and [59]; Katrivanos et al. [23]; Georgiadis et al. [56]; and own field work). The Pelagonian zone is in an underthrust position relative to the Cretaceous carbonate platform of the Vardar zone [56] (B–B´). Imbricated ophiolite and Jurassic limestone are exposed in a window extending from Margarita to Veria. Metamorphosed Pelagonian limestone is exposed in the Gandach antiform of the Paikon sub-zone near Livadia. The tectonic section A–A´ is shown in Fig. 4b. The formations between the Gandach marble and the Theodoraki limestone is a composite mélange.

**Plate 1:**
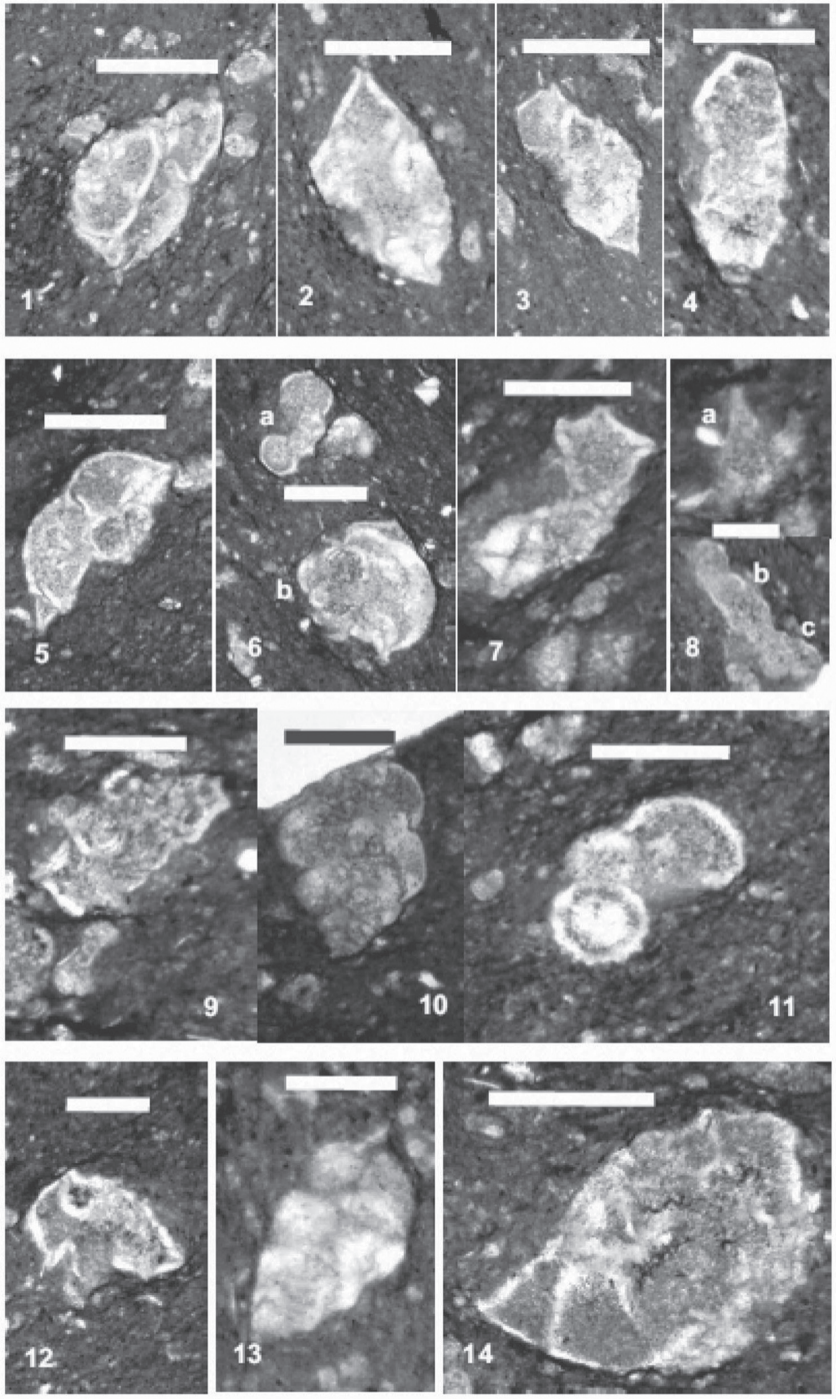
Scale bars: Figs 1–14 Fig. 1. *Contusotruncana fornicata* (Plummer). Fig. 2. *Globotruncanita stuarti* (De Lapparent). Fig. 3. *Globotruncana arca* (Cushman). Fig. 4. *Globotruncana linneiana* (d’Orbigny). Fig. 5. *Radotruncana subspinosa* (Pessagno). Fig. 6. a) *Rugoglobigerina hexacamerata* Brönnimann, b) *Radotruncana subspinosa* (Pessagno). Fig. 7. *Globotruncana aegyptiaca* Nakkady. Fig. 8. a) Schackoina sp., b) *Ventilabrella glabrata* (Cushman), c) *Rugoglobigerina hexacamerata* Brönnimann. Fig. 9. *Globotruncana lapparenti* Bolli. Fig. 10. *Heterohelix dentata* (Stenestad). Fig. 11. *Rugoglobigerina rugosa* (Plummer). Fig. 12. *Globotruncana rosetta* (Carsey). Fig. 13. *Heterohelix carinata* (Cushman). Fig. 14. *Globotruncanita atlantica* (Caron).

Sheared Eohellenic ophiolite occurs on top of Pelagonian carbonates in contact with disrupted Cretaceous limestones (Table 1b 1 and 2), along the western border of the Vardar zone, for example, near Panagitsa and Arnissa (Fig. 6) [55] and southwards near Pyrgi–Kato Grammatiko and west of the Vermio mountains [56] (Fig. 6). West verging imbricated thrust faults characterise this western boundary of the Vardar zone, from the Dinarides through the Hellenides (in Jacobshagen [53]). The base of the imbricates is Eohellenic ophiolite and the Triassic–Jurassic carbonate platform of the Pelagonian zone which is covered by disrupted ophiolite followed by schistose pyroclastic units interleaved with slices of radiolarian cherts, volcaniclastic and chloritic marble layers. This tectonic transition between Pelagonia and the western edge of the Vardar zone is shown by Sharp and Robertson [6] in the Arnissa area (Fig. 6): a ~500-m-thick succession of imbricated ophiolite mélange. This succession is topped off by limestone debris with rudists and planktonic foraminifera, *Globotruncana* [55] (Table 1b 3) (Plate 1). In agreement with these observations, we underscore that the contact between the Vardar and Pelagonian zone is a thrust-fault-zone (see Discussion and conclusions).

**Plate 2:**
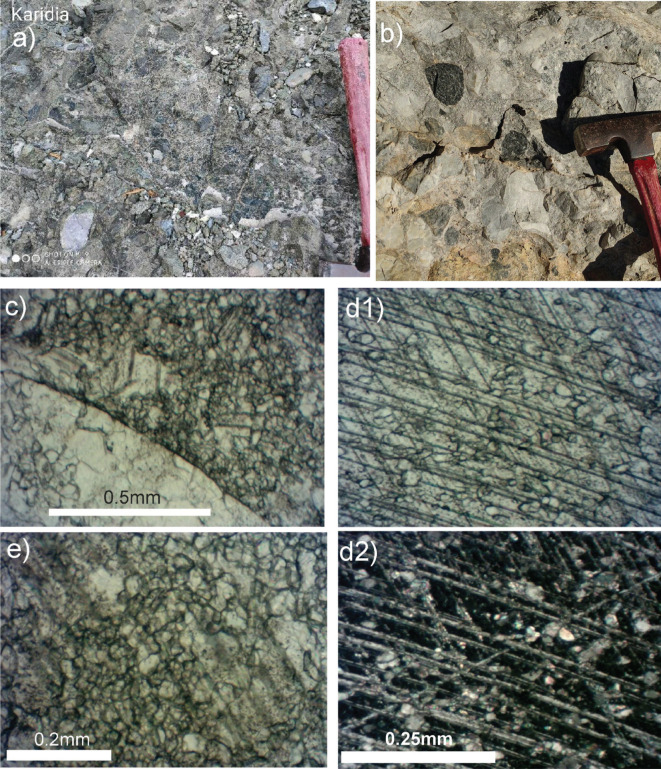
a. Field photo: breccio-conglomeratic ophiolite mélange in west Almopias, near Karydi. b. Field photo: breccio-conglomeratic carbonate mélange in west Almopias near Nisi. c. Photomicrograph: rounded grain of limestone and adjacent matrix of micro-breccia without cement. d1, d2. Photomicrographs: neomorphic calcite (parallel and crossed nicols) in the matrix of 2b, showing palimpsest relic matrix grains and twinning planes. e. Photomicrograph: matrix of 2b showing initial palimpsest texture of growing neomorphic calcite in the matrix with recognisable twin planes.

Although Cretaceous carbonates have been supposed to *transgressively* overlie laterite and serpentinite [6,47,55], we are of the opinion that the inferred transgressional conglomerates are cataclasites (Plate 2a,b) and that orthoconglomerates [57] that could substantiate a marine transgression have not been verified (see Discussion and conclusions). Furthermore, the Cretaceous limestones of the Vardar zone are in tectonic contact with the subjacent allochthonous substrate even where post-Eohellenic laterite is found along the contacts. The circumstances here are similar to the Northern Sporades where a *sedimentary* contact of the Cretaceous Carbonates with their original substrate is nowhere to be found [10].

#### Tectonic windows in west Almopias

Serpentinite and ophiolite–carbonate mélange crop out through the Cretaceous limestone cover in tectonic windows along a narrow, elongated zone of north–south striking faults, extending from Kerassia–Karydia–Kedronas [55,58] to Ano Grammatiko [18,56] (Fig. 6). Extensive exposures consist of ‘conglomeratic’ rocks [55], which in our opinion are cataclasites (see Plate 2 and Discussion and conclusions). The ‘conglomeratic’ rocks contain Triassic and Jurassic carbonates as well as limestones ranging in age from Cenomanian to Turonian (Table 1b 2) and overlie Pelagonian serpentinite [55]. Near Nisi and Karydia (Fig. 6) these cataclasites (Plate 2a,b) occur below Campanian limestone (Table 1b 4) (Plate 1). At its base, this succession contains olistolith marbles of Triassic–Jurassic age and overlie white micaceous Triassic marbles in suggested *transgressional* contact [55]. We dispute a transgressional origin of the Kedronas–Nisi ‘conglomerate’ (see discussion on pseudo-conglomerates). The tectonic windows exposing underthrust Pelagonian ophiolite rocks can be followed in west Almopias from the north near Karydia to the Vermion area [56] (Fig. 6, see section B-B´).

#### Pelagonian ophiolite exposures of central Almopias

An extensive imbricated belt of ophiolite mélange some 50 km long and 5–10 km wide can be traced from the Lyki–Klissochori area [55,59] to the Naousa and Veria areas (Fig. 6) [18,56,60]. The mélange is interleaved with slices of marble and Jurassic carbonates, which we agree, are of Korab–Pelagonia origin [56,61] (Table 1b 6 and 7–7.2). The carbonates contain an Oxfordian–Kimmeridgian reefal fauna, including *Cladocoropsis* sp. of Late Jurassic age [59]. As pointed out above, this is a typical Kimmeridgian–Tithonian reef facies of the Pelagonian zone [21] (Table 1a 7–8) that had been overthrust by Eohellenic ophiolite during the Early Cretaceous. In the Vardar zone, the Pelagonian ophiolites are locally interleaved with sericitised basalt schist (Lyki) (see Geochemistry) and are in an underthrust position beneath ‘conglomeratic’, ophiolitic mélange and upper Cretaceous carbonates (east of Margarita, Fig. 6) (Table 1b 7).

In accord with the previously cited researchers and the described geology, we support the opinion that the ophiolites and upper Jurassic carbonates found in the west and central Vardar sub-zones are tectonically inherited from the underthrust Pelagonian plate (Fig. 4b).

#### Eastern Almopias and Paikon units

A noteworthy difference between the eastern and western units of the Vardar zone is that the eastern Almopias and the Paikon units appear to be devoid of serpentinite which we corroborate from Tranos et al. [62]. Serpentinite, however, probably exists at depth (Fig. 4b), because further north in an area known as Ano Garefi serpentinised peridotite is exposed below basalt [60]. The mélanges of the Nea zoi-Vryssi-Meglenitsa and Krania units (Figs 4b and 6) are composed of dolerite, pillow basalt and tuff and contain upper Jurassic–lower Cretaceous radiolarite [59], with a relict Cretaceous cover (Table 1c 1.–1.2). Slices of Triassic lavas and radiolarites [63] (Table 1c 3 and 4) and upper Cretaceous arenites are also incorporated into the foliated matrix of the mélange of the Krania–Vryssi units [60]. The ‘*ophiolite related*’ mafic units, ‘*ophiolite nappe*’ and ‘*Meglenitsa Ophiolite*’, reported in Sharp and Robertson [6] (from Sharp and Robertson [64] and Sharp and Robertson [65]), in our opinion are not ophiolites s. str. but consist of ocean floor or arc basaltic rocks (see Geochemistry).

#### The Paikon antiform, a Pelagonian window: Katrivanos et al. [23]

The Theodoraki limestone is the youngest formation of the Paikon antiform [23]. This limestone is part of the Cretaceous carbonate platform that covers the entire Vardar zone, and is composed of a wide range of neritic to reefal facies (Table 1b and Table 1c Theodoraki unit). The platform is in tectonic contact with a pile-up of SW dipping slices of Theodoraki limestones and slices of volcano-sedimentary rocks including radiolarites, tuffites and lava, and Triassic-Jurassic Marble and schist of Pelagonian origin [17]. Katrivanos and others [23] corroborate that the tectono-stratigraphic sequence is composed of volcano-clastic rocks together with limestones of Middle to Late Jurassic age, based on micro and macro-faunas including *C. mirabilis* (Griva–Kastaneri formation Fig. 4b, Fig. 6) (Table 1c Griva–Khromni units). The volcano-sedimentary slices are on top of Triassic–Jurassic Gandatch marbles and schists (Fig. 6). All the volcanic material of this series is *strongly mylonitised in discrete, narrow shear zones* related to mylonitic foliation [23]. The carbonate rocks are mylonitised, near the contacts with tectonically overlying volcano-sedimentary slices, for example, at Kastaneri [23]. Our investigations corroborate the above observations, which lead us to interpret the volcano-sedimentary formations in the substrate of the Theodoraki limestone as a composite **allochthonous mélange complex** in which slices of volcanic and sedimentary rock-units can be individually distinguished.

In contrast to the above, the Paikon unit has been depicted [64] to consist of a contiguous sedimentary, stratigraphic, succession extending from the Triassic to Cretaceous time only interrupted by two unconformities, an Oxfordian and a Cenomanian.

We share the opinion that the **Paikon is an antiform and a Pelagonian tectonic window** [23], and that the Paikon unit of the Vardar zone was most probably part of a **volcanic island arc complex** [3,8,15,17,60]. Our mutually envisioned island arc scenario evolved as the eastern Vardar Ocean subducted north-eastwards towards the margin of the European continent, which initiated supra-subduction arc volcanism [8,17,60]. This was accompanied by back-arc spreading (3,5), represented by the Guevgueli ophiolite complex (Fig. 4b) [61,66–68].

### Geochemistry

**Figure 7:**
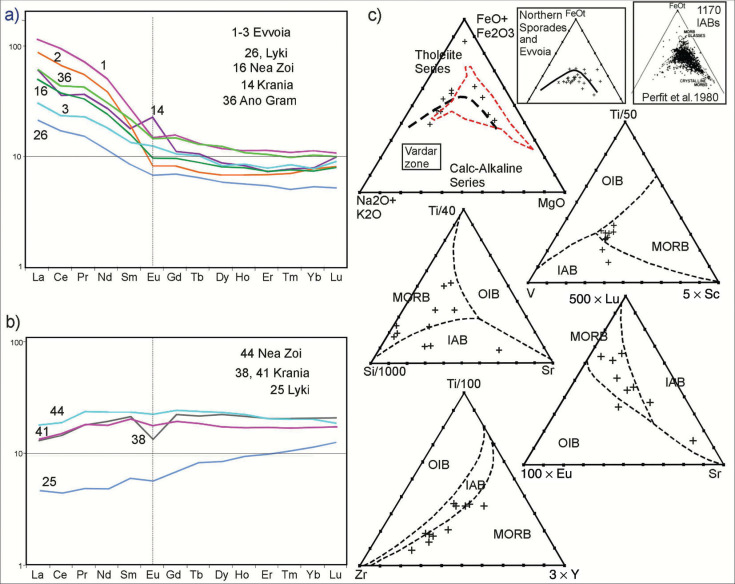
Chondrite-normalised REE and ternary discrimination diagrams. a) LREE enriched samples, probably IABs. b) Flat REE and LREE depleted samples, most likely MORBs (see text). c) Discrimination diagrams: Vardar zone data (AFMs are also shown for Evvoia and the Northern Sporades). The AFM from Perfit and others [70] shows the plots of 1170 IABs (the dashed red line area in the Vardar diagram, encompassing only a few of the Vardar meta-basalts).

Meta-basalts from the Vardar zone and from northern Evvoia have been analysed for their major, minor and trace element contents. The analytical methodologies used were inductively coupled plasma-optical emission spectrometry (ICP-OES) and ICP-mass spectrometry (MS) analyses by four-acid (hydrochloric, nitric, perchloric and hydrofluoric) digestion which are ‘near total’ digestions. *Instrumental neutron activation analysis* (*INAA*) analysis yields total metals – samples are encapsulated and irradiated in a nuclear reactor. After a suitable decay, samples are measured for the emitted gamma ray fingerprint. Some previous analyses are shown from the Northern Sporades [10]. The analytical results are shown in Tables 2a and 2b. Rare earth element (REE) plots and ternary discrimination diagrams (Fig. 7) have been drafted for the purpose of ascertaining basalt origins (after Pearce and Cann) [69–71]. Two serpentinised peridotites associated with basalts and radiolarian cherts from Pelagonian ophiolites of Evvoia were previously analysed [10] (Table 2a).

**Table 2a.** Major and trace elements for the Vardar zone (FICP and FMS)

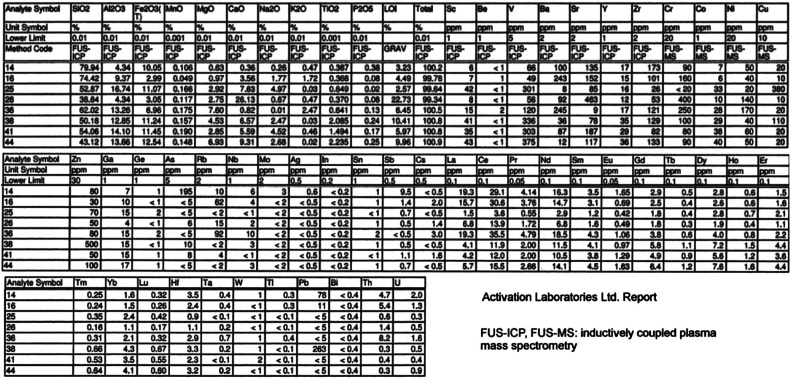

**Table 2b.** Major and trace elements for Evvoia and the Northern Sporades (same analytical information as in Table 2a)

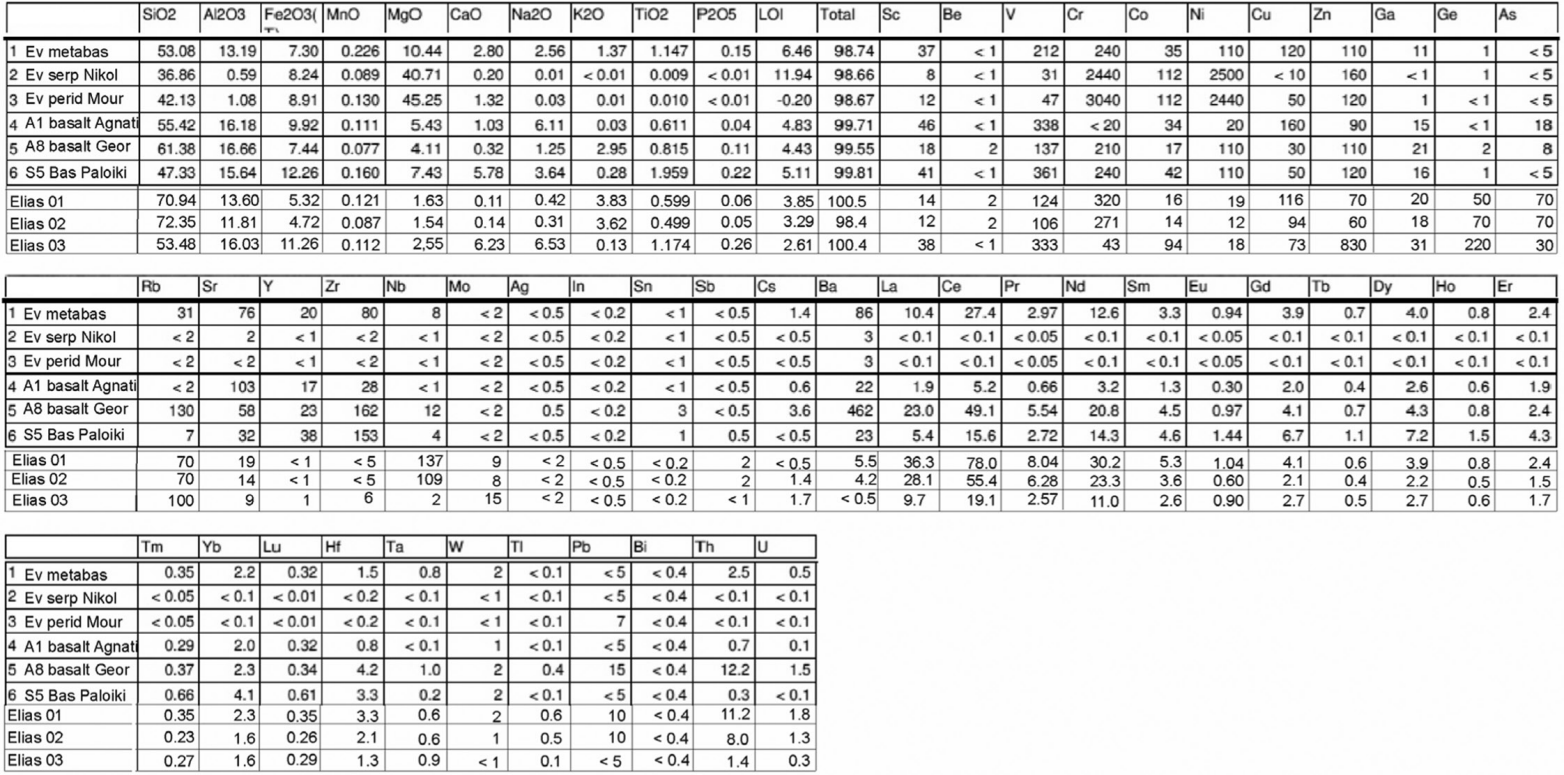

The meta-basalts of the Vardar zone and the Northern Sporades occur in mélanges and they are sheared and sericitised and strongly weathered, which may have caused contaminations with adjacent rocks, making unambiguous differentiation between mid-ocean ridge basalts (MORBs) and island acr basalts (IABs) additionally more problematic than it intrinsically is anyway (as Perfit and others, [70], point out). None of the analyses (Table 2a) have abnormal chromium (Cr) or nickel (Ni) contents which excludes serpentinite contamination (compare Cr and Ni Table 2b samples 2–3).

The REE plots are typical for basalts [69,70,72,73] (Fig. 6a and b). They depict light REE (LREE), enhanced patterns, associated with IABs, and flat LREE-depleted patterns associated with MORB origins. An almost identical array of REE plots have been ascertained for the Northern Sporades where the present authors had drawn the conclusion that MORBs and IABs had been tectonically mixed in the mélanges of an extensive thrust-fault zone (Fig. 7) [10]. As in the Northern Sporades, the REE-plots drafted for the Vardar zone indicate the presence of both IAB and MORB (Fig. 7a and b). Discrimination diagrams (Fig. 7c) also indicate the ambiguous situation that MORBs for samples in one diagram correspond to IABs in another.

Following Perfit and others [70] we have additionally checked that according to Perfit [70] there are distinguishing differences in potassium (K), titanium (Ti) and total iron (Fe)wt.% concentrations in IABs and MORBs: MORBs having <0.25 potassium oxide (K2O), IAB having >0.25 K2O; IAB having <1.2 titanium oxide (TiO2) and >6–15 total Fe. The results of this query, using data from Tables 2a and 2b, it appears that most of our samples are IABs but there are numerous ambiguities which, presumably, are caused by tectonic mélange mixing.

The analyses of the basalts from the Eohellenic ophiolite of Evvoia and those of the Elias complex are incorporated in the REE and atomic force microscopy (AFM) diagrams (Fig. 7a and c) (Table 2b) and they indicate MORB and IAB affinities.

## Discussion and conclusions

### The composite tectono-stratigraphy of eastern Pelagonia and the Vardar zone in context with the previously related geology

**Figure 8:**
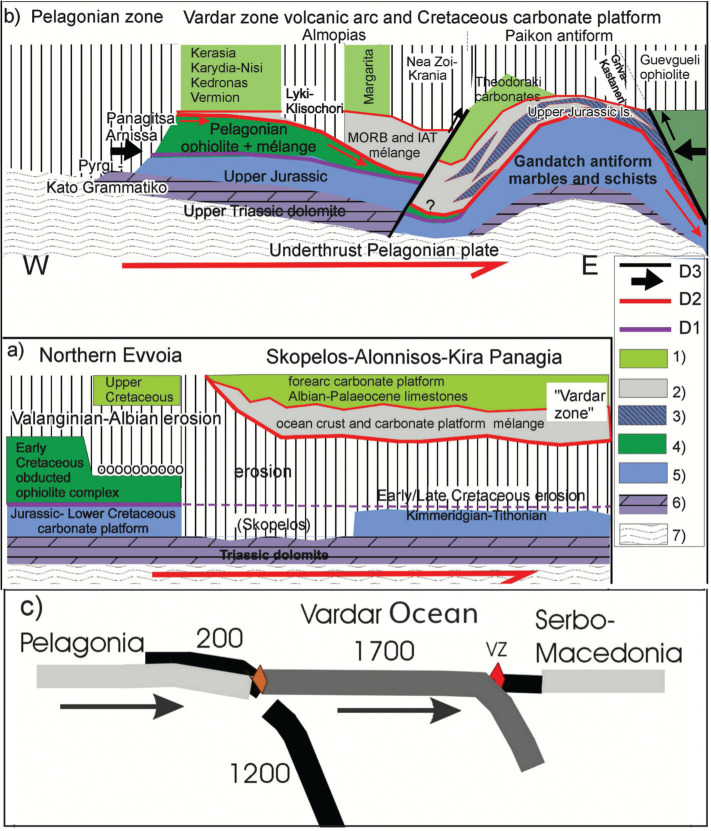
Composite tectono-stratigraphic synopsis: a) Evvoia and the Northern Sporades were overthrust by the Eohellenic ophiolite, which was subsequently deeply eroded and transgressed by ~Cenomanian conglomerates. On the Northern Sporades, the ophiolite and Lower Cretaceous had been removed by erosion before being underthrust (D1–D3) beneath the Vardar zone sheet during the Paleocene time. b) Likewise, the Vardar zone was underthrust by Pelagonia, which carried remnants of Eohellenic ophiolite and possibly Cenomanian orthoconglomerates. c) Schematic section through the Vardar Ocean between Pelagonia and Serbo-Macedonia indicating the widths (km) of oceanic lithosphere (see seismic tomography). Legend: 1) Cretaceous and Paleocene carbonates. 2) mélange including Triassic radiolarite and basalt, pyroclastic rocks and carbonate slices. 3) Upper Jurassic (Pelagonian slices) and lower Cretaceous Theodoraki carbonate slices. 4) Pelagonian ophiolite s. str. 5) Pelagonian Jurassic carbonates. 6) Pelagonian upper Triassic dolomite. 7) Crystalline basement of Pelagonia. D1–D3 deformations (see text).

Pelagonia consists of a Palaeozoic–Middle Triassic basement covered by a carbonate platform over which a 200 km-wide ophiolite sheet of west Vardar Ocean lithosphere had been obducted (Fig. 8a–c). The 1700-km wide eastern Vardar Ocean subducted beneath the Vardar zone (vz) during the Late Jurassic through Cretaceous time (Fig. 8c). Figure 8a and b suggests that Pelagonia together with obducted Eohellenic ophiolite collided with the Vardar zone and underthrusts the Cretaceous–carbonate platform and its volcano-sedimentary substrate (Fig. 8b). As Pelagonia continued to advance it underthrust the Guevgueli complex and crashed with Serbo-Macedonia (Fig. 8b and c).

### Major deformations

Three major episodes of tectonic deformation, D1–D3, affected the Pelagonian and Vardar zones; each dominated by a major time-transgressive thrust fault complex (Fig. 8a and b). D1 and D2 occur in both study areas; D3 is evident in the Vardar zone but has not been verified in the Northern Sporades (Fig. 8a and b). (Our D1–D3 indices do not correspond with those of previous researchers [17,23,74].)

Deformation D1 is Eohellenic (Fig. 8a), involving the westward obduction of the Eohellenic (west Vardar Ocean) ophiolite onto eastern Pelagonia (Fig. 8c). Post-D1 erosion, especially prominent in Skopelos, is suggested to have been caused by widespread Pelagonian uplift as the sinking Vardar (1200 km) slab broke off in the post-Valanginian time (Fig. 8c).

Deformation D2: Pelagonia, the trailing edge of the eastward subducting Vardar plate, crashed with and underthrust the Vardar arc, causing shearing, myolonitisation and imbrication between the overriding Cretaceous carbonate platform including its volcano-sedimentary substrate. Greenschist and high-pressure/low-temperature (HP/LT) metamorphism described by Katrivanos et al. [23] can be attributed to D2.

Deformation D3 corresponds to the compression effected by the crash of the Pelagonian plate with Serbo-Macedonia, which caused folding in the Vardar and Pelagonian zones whereby the Paikon antiform is the most prominent (Fig. 8b). An analogical antiform has not been observed in the Northern Sporades but could be sought in the central Aegean Sea (Fig. 8a). Shear–stress caused by the crash produced the youngest thrust faults in the flanks of the Paikon antiform (D3 in Fig. 8b) and most probably rejuvenated older faults, including numerous subordinate imbrication thrusts (Fig. 4b), described in Mercier and Vergely [17], Kilias et al. [74] and Katrivanos et al. [23].

### Pseudo conglomeratic mélange of Kedronas, Nisi and Karydia

The breccio-conglomeratic, cataclastic rock complex that contains abundant rounded clasts occurs incorporated in an extensive fault zone mélange in the west Almopias unit between Karydia and Ano Grammatiko (Plate 2a, b) (Fig. 6 pseudo conglomeratic mélange). In the Nisi-Karydia area the cataclasites are in tectonic contact with Campanian limestones on top (Plate 1) (Table 1b 4.1) and Pelagonian ophiolite at the base. We regard the cataclasites as matrix supported parabreccias composed of poorly sorted >2 mm, rounded to angular clasts (Plate 2a, b). The clasts either consist predominantly of marbles, elongated pieces of sericitic calc-schists and dark micritic limestones (Plate 2b) or are chaotic mixtures of carbonate and ophiolite clasts (Plate 2a). Viewed under the microscope, the matrix is a chaotic breccia of calcite and carbonate grains that are not bound by interstitial pore cement [75] but by insular patches of aggrading neomorphic sparry calcites that had grown amid the much smaller angular granules of the matrix (Plate 2c, d, e). Crushed neomorphic calcite occurs in the matrix inherited from earlier stages of shearing. The neomorphic calcite, unlike cement, exhibits irregular boundaries and palimpsest, relic-matrix texture (Plate 2 d–e). The neomorphic calcites exhibit residual stress, indicated by crossing twins, stopping twins, twin thickening and bending, which appears in low temperature stress regimes below 200°C [76,77]. Neomorphism had most likely taken place in a dry sub-metamorphic environment (Folk in Bathurst [75]).

It is suggested that the larger components underwent rounding and grain-reduction by granulation from the decimetre to centimetre scale to the microscopic micron scale, which is not unusual in tectonic breccias in which the fragments may be worn down and rounded by tectonic grinding [78–80].

We dispute that this rock complex had a transgressional origin (Mercier and Vergely [55] and Mercier in Sharp and Robertson [6]) because it does not display the most important characteristics that marine conglomerates should have: clast–clast support and diagenetic cement [75]. On the contrary the clasts are matrix supported and the grains have not been diagenetically cemented. In our opinion the ‘parabreccio-conglomerate’ formed as Pelagonia underthrust the Vardar zone during Paleocene time (D2 above).

#### The collision of two Cretaceous carbonate platforms

It should be taken into consideration that some remnants of the well-documented Cretaceous Pelagonian carbonate platform (Fig. 8a), may have been subducted (‘piggy-backed’) beneath the Cretaceous carbonate platform of the west Almopias, at the latest during the Paleocene time, and thus possibly inherited Pelagonian-orthoconglomerates could occur in the mélanges beneath the Vardar zone (e.g., Vermion: Photiades et al. [47]).

#### New Paleogeography

**Figure 9:**
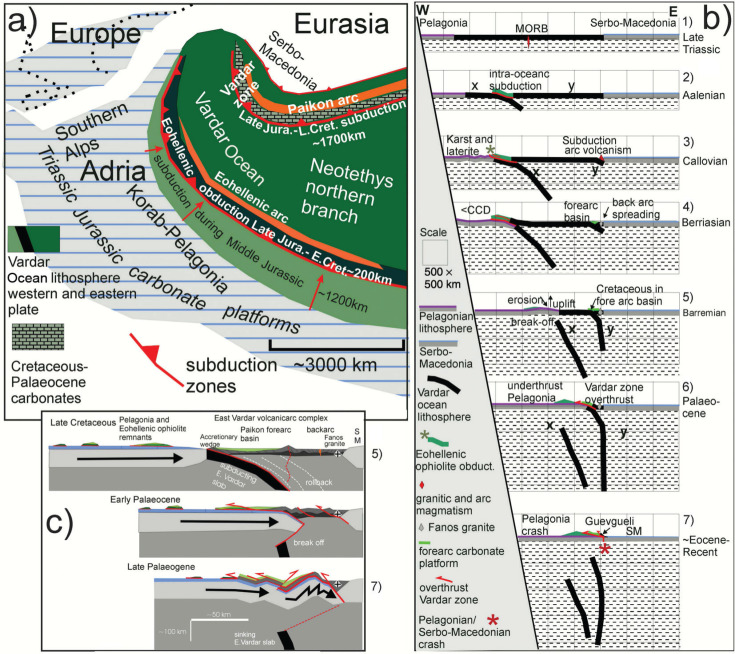
Paleogeography and time-lapse cartoons. a) The Vardar Ocean was situated between two passive margins, continental Adria (including Korab-Pelagonia) and Serbo-Macedonian Europe. Early Middle Jurassic intra-oceanic subduction led to the demise of about 1200 km of the Vardar lithosphere and to the obduction of about 200 km of the Eohellenic ophiolite onto eastern Pelagonia. Subsequently, about 1700 km of the Vardar Ocean lithosphere subducted beneath the Paikon (east Vardar) island arc, followed by the crash of eastern Pelagonia with the island arc, and finally (c) to the collision of Pelagonia with Serbo-Macedonia. b) This time-lapse cartoon shows the demise of the Vardar Ocean in seven stages. Pelagonia and Vardar Ocean lithosphere move NE toward, relatively autochthonous, Eurasia. Pelagonia and Vardar Ocean lithosphere move NE toward, relatively autochthonous, Eurasia. The Vardar Ocean slabs are shown as they reach their present position shown in Fig. 3c. It is important to note that the Earth’s curvature has been neglected in the graphic. This creates distortion in the lower mantle making it appear wider than it should be. Time schedule of subduction 1) The Vardar Ocean existed during Late Triassic time verified by radiolarians associated with pillow basalt (Table 1a Carnian-Norian). 2) Intra-oceanic subduction was in progress around Toarcian to Aalenian time (180–170 Ma), based on the metamorphic age of subduction-zone amphibolite mélange [41,42]. Relative plate motions, however, had already changed from divergence to convergence, during the Late Triassic, testified by the subsidence of the Rhaetian–Sinemurian peritidal carbonate platform and change to the subtidal platform of Pliensbachian and Toarcian time [33] (Table 1a Rhaetian-Pliensbachian). Subduction of slab (x) continued through the Middle Jurassic, verified by late Middle Jurassic radiolarians in ophiolite mélange in Evvoia [34,36]. 3) Platform uplift, erosion and bauxite deposition occurred during the Callovian [35,44], presumably due to the crash of the Eohellenic arc with the Pelagonian platform (Callovian unconformity [35,44], causing upwarping of the carbonate platform. This stress communicated across the east Vardar Ocean causing subduction between east Vardar and Serbo-Macedonia. 4) As the Eohellenic ophiolite advanced, the carbonate platform subsided below the CCD during the Kimmeridgian–Berriasian time while back arc spreading was taking place in Guevgueli. 5) The final Eohellenic ophiolite emplacement takes place about Valanginian time. The west Vardar slab x breaks off and sinks, the Pelagonian platform rises and deep (post-Eohellenic) erosion of the Eohellenic nappe takes place. The Cretaceous carbonate platform evolves on top of volcanic debris of the forearc basin and accretionary wedge. The east Vardar slab (y) continues to subduct. 6) Pelagonia crashes with the arc, underthrusts the Cretaceous carbonate platform and volcanic arc, and the Guevgueli back arc basin. 7) Pelagonia crashes with Serbo-Macedonia while the Vardar slab breaks off and subsides. c) The cartoon shows the final episode of Vardar Ocean subduction. Pelagonia crashes and underthrusts the arc and the Vardar slab breaks off. Pelagonia collides with Serbo-Macedonia, which initiates folding and renewed thrust faulting.

From the evidence presented here and from seismic tomography it is postulated that the Vardar Ocean subducted along two subduction zones (Fig. 9a). The western intra-oceanic subduction zone evolved about the Toarcian to Aalenian time, based on radiometric ages of amphibolites in sub-ophiolite mélanges, and continued to subduct through the Middle Jurassic verified by late Middle Jurassic radiolarians in the sub-ophiolite mélange in Evvoia [34,36,38] (Table 1a 11.2 and 12). A supra-subduction volcanic arc evolved during the Middle Jurassic, documented by the Elias complex of northern Evvoia (Fig. 4a) which presumably was part of a more extensive supra-subduction ‘Eohellenic arc’ (Fig. 9a) [34]. The beginning of the Eohellenic obduction, is suggested to have begun during the Bathonian time together with the Callovian upheaval [44] and the eastward subduction of the eastern Vardar Ocean (Fig. 9b 3). The Vardar, supra-subduction, volcanic island arc and the spreading Guevgueli back arc ophiolite complex evolved during the (Middle?) Late Jurassic and Cretaceous times. We envisage a Paikon forearc basin, rimmed by an accretionary wedge like that shown in Saccani et al. [67] in which the basin floor was initially covered by (volcanoclastic) basalt without carbonates during the lower Middle Jurassic. To the best of our knowledge, a Jurassic carbonate platform did not evolve in the Vardar zone. We suggest that Jurassic–early Cretaceous volcanoclastic deposits accumulated on the flanks of the Vardar volcanic arc and became the substrate of carbonate accumulation beginning in Aptian time. Investigations of the Guevgueli back arc basin have not disclosed relicts of a carbonate platform [67].

### The Cretaceous forearc carbonate platform of the Vardar zone

The Cretaceous Vardar zone-carbonate platform is envisaged to have evolved over the late Jurassic–early Cretaceous volcanoclastic substrate of the forearc basin (Fig. 9a) (fig. 12 in Saccanni et al. [67]).

The earliest recorded Cretaceous limestones in the Vardar zone are of Aptian age (Table 1b 4.2, Table 1c 6.1). The bio facies indicate a reefal to inner neritic environment having had depths of between 10 and 50 m [81]. These limestones are in the west Almopias sub-zone (Fig. 4b) and may have been deposited near or on the accretionary wedge of the forearc basin [67]. The verified bio facies indicate that patch reef and neritic environments existed side by side through the Cenomanian, Santonian, Campanian, and Maastrichtian times (Table 1b West Almopias) (Plate 1). The deeper neritic platform facies occur eastwards in the central and east Almopias sub zones, ranging in age from the Cenomanian to Maastrichtian (Table 1b–1c Central and East Almopias). The bio stratigraphic succession in the Theodoraki limestone formation begins with Cenomanian/Turonian reef facies that may represent a fringing reef along the outer slopes of the arc. Inner neritic facies deepen upwards, from the Campanian to Maastrichtian times (Table 1c 5 Theodoraki unit). Late Maastrichtian flysch signals the demise of the Cretaceous carbonate platform of the Vardar zone.

From the aforementioned, a tentative picture of the platform architecture can be discerned: it was a subsiding environment in which about 500 m of carbonates accumulated (‘carbonate factory’ [25]) during about 60 Ma between the Aptian and Maastrichtian times [55,59]. Reefs evolved during the Early Cretaceous along an outer western accretionary wedge and an inner eastern high where fringing reefs on the outer slopes of the Paikon volcanic arc interdigitated outer neritic carbonate facies in the central basin.

#### Seismic tomographic images of the mantle below the Hellenides

We have interpreted the perturbations beneath Hellenides as sunken Vardar Ocean lithosphere which indicate two episodes of subduction [10] (Fig. 3c).

The vertical section (Fig. 3c) shows that the leading edges of each slab has sunk to a depth approaching 2000 km. Presently, the trailing edge of the western slab (x in Fig. 3c) is about 900 km below the Earth’s surface and the trailing edge of slab (y) is about 400 km below the surface. These are the depths to which the slabs have sunk since their breakoffs. In estimating the width of a slab, however, one must consider that a subsiding lithospheric plate certainly undergoes compression and deformation which can make width-estimates inaccurate (Fig. 3e). The seismic tomographic images are, nevertheless, presently the best possible way to estimate the onetime width of the subducted oceanic lithosphere, which we estimate to have been about 3000 km [determined by adding together the lengths of the slabs (x + y) ~1200 + ~1700 and adding, to that sum, the width of the obducted Eohellenic ophiolite sheet, which has been assumed to be about ~200 km (Fig. 8c)]. However, 3100 km is the composite width, not necessarily the surface width that the Vardar Ocean had at any one time. We do not know when the ocean ridge stopped spreading: subduction and ocean spreading at the ocean ridge could have taken place simultaneously.

The western slab (x) is supposed to have broken off and began sinking after the Eohellenic ophiolite had been emplaced during Valanginian time. The eastern Almopias slab (y) is supposed to have broken off after Pelagonia crashed and underthrust the Vardar zone-carbonate platform and volcanic arc complex.

### Seismic tomographic model

Our model (Fig. 9) postulates that the Vardar Ocean was about 3000 km wide and bordered on Adria in the west. This means that both the microplate Adria and the vaguely attached African plate, were situated 3000 km further southwest during the Early Jurassic time as the Atlantic Ocean and the Alpine Tethys began spreading (e.g., Schmid et al. [2]; Scherreiks et al. [33]). This implies that Pelagonia, the eastern edge of Adria, moved about 3000 km northeast towards the European continent (Fig. 9b) while the Atlantic spread.

The ~3000 km wide Vardar Ocean is supposed to have subducted/obducted, between the (~Sinemurian) Aalenian time (175 Ma) and the Paleocene time (~65 Ma), roughly a time span of 175–65 = 110 Ma. Subduction rates of the oceanic slabs are estimated to range from about 3 cm/year (= 30 km/1 Ma) in the upper mantle to about 1 cm/year in the lower mantle [82]. Simple calculations show that at a rate of 30 km/1Ma, a 3300 km wide ocean would subduct in 110 Ma; and a 3000 km wide ocean could subduct in 110 Ma at a rate of ~2.7 cm/a.

In our example, we also consider that the trailing edge of slab x sank 900 km since breaking off after the Valanginian time, and the trailing edge of slab y sank about 400 km since its breakoff in the ~Paleocene.

Sinking rates are lower in the mantle below 300–500 km, and in the lower mantle slab subsidence eventually approaches zero [83,84]. We have previously estimated [10,11] that in using an **average** subsidence rate of 0.68 cm/year, one arrives at a Hauterivian break-off date for slab x (900 km/6.8 km/Ma ~132 Ma), and Late Paleocene as the break-off time of slab y (400 km/6.8 km/Ma ~59 Ma), which we believe corresponds to the known facts and is well in the range of plausibility.

## Summary

The demise of the once over 3000-km wide Vardar Ocean has been reconstructed from field investigations of its remnants in its onetime peripheries, and from seismic tomographic images of its remnants in the mantle below the Central Hellenides. On its southwestern side the Vardar Ocean bordered on the Pelagonian–Adriatic plate, which was covered by a vast carbonate platform [31] that evolved from a peritidal realm during Norian–Sinemurian to a drowned platform during the Tithonian–Berriasian time. In the northeast the Vardar Ocean bordered on Serbo-Macedonia of the European plate, where, during the Late Jurassic a supra-subduction volcanic island arc and back-arc complex emerged. A forearc reef and a shallow marine carbonate platform accumulated on top of a Jurassic–early Cretaceous volcano-clastic substrate from about the Aptian through the Maastrichtian time.

The closure of the Vardar Ocean occurred in temporally overlapping episodes: one episode of ophiolite obduction and two episodes of intra-oceanic subduction.

1. During the Middle Jurassic time a 1200-km slab of west Vardar lithosphere subducted eastwards beneath the ‘Eohellenic’, arc, while a 200-km wide slab obducted westwards onto Pelagonia between the Callovian and Valanginian times.
2. A 1700-km wide slab of east Vardar lithosphere subducted eastwards beneath the Vardar zone arc-complex during the Late Jurassic through the Cretaceous time and subsequently Pelagonia underthrust the Cretaceous carbonate platform during the Paleocene.

In the greater framework of plate tectonics, the subduction of the Vardar Ocean occurred simultaneously with the spreading of the Atlantic Ocean and the opening of the Alpine Tethys, while the Hellenides moved about 3000 km toward the northeast.

In the light of the present contribution, future research concerning the evolution of the Cretaceous carbonate platform of the Vardar zone could advance our knowledge of the facies distributions and architecture of the Paikon fore arc basin. Another point of interest is the seismic tomography and the demise of the Guevgueli back arc since the Paleocene time.

## Data Availability

The datasets generated during and/or analysed during the current study are available from the corresponding author on reasonable request.
